# The statistical design and analysis of pandemic platform trials: Implications for the future

**DOI:** 10.1017/cts.2024.514

**Published:** 2024-10-15

**Authors:** Christopher J. Lindsell, Matthew Shotwell, Kevin J. Anstrom, Scott Berry, Erica Brittain, Frank E. Harrell, Nancy Geller, Birgit Grund, Michael D. Hughes, Prasanna Jagannathan, Eric Leifer, Carlee B. Moser, Karen L. Price, Michael Proschan, Thomas Stewart, Sonia Thomas, Giota Touloumi, Lisa LaVange

**Affiliations:** 1 Duke University, Durham, NC, USA; 2 Vanderbilt University Medical Center, Nashville, TN, USA; 3 University of North Carolina at Chapel Hill, Chapel Hill, NC, USA; 4 Berry Consultants, Austin, TX, USA; 5 National Institute of Allergy and Infectious Diseases, Bethesda, MD, USA; 6 National Heart, Lung, and Blood Institute, Bethesda, MD, USA; 7 University of Minnesota, Minneapolis, MN, USA; 8 Harvard T.H. Chan School of Public Health, Boston, MA, USA; 9 Stanford University School of Medicine, Stanford, CA, USA; 10 Eli Lilly and Company, Indianapolis, IN, USA; 11 University of Virginia, Charlottesville, VA, USA; 12 RTI, Raleigh, NC, USA; 13 National and Kapodistrian University of Athens, Athens, Greece

**Keywords:** Clinical trials, platform trials, pandemic preparedness, research design, statistical analysis

## Abstract

The Accelerating COVID-19 Therapeutic Interventions and Vaccines (ACTIV) Cross-Trial Statistics Group gathered lessons learned from statisticians responsible for the design and analysis of the 11 ACTIV therapeutic master protocols to inform contemporary trial design as well as preparation for a future pandemic. The ACTIV master protocols were designed to rapidly assess what treatments might save lives, keep people out of the hospital, and help them feel better faster. Study teams initially worked without knowledge of the natural history of disease and thus without key information for design decisions. Moreover, the science of platform trial design was in its infancy. Here, we discuss the statistical design choices made and the adaptations forced by the changing pandemic context. Lessons around critical aspects of trial design are summarized, and recommendations are made for the organization of master protocols in the future.

## Introduction

On 17 April 2020, the Therapeutic-Clinical Working Group of the Accelerating COVID-19 Therapeutic Interventions and Vaccines (ACTIV) initiative was established. A Master Protocol Working Group was tasked with quickly developing protocols to evaluate selected treatments in both inpatient and outpatient populations. The initial goal was to launch at least three master protocols within 4–5 months. Each master protocol was expected to describe a trial designed to leverage the same infrastructure for evaluating multiple treatments simultaneously, allowing new agents to enter, for some agents to graduate from Phase II to Phase III, while others may be discontinued early. These are known as platform trials [1]. A total of 11 master protocols were developed, of which two remain ongoing at the time of this writing (ACTIV-4 Host Tissue and ACTIV 6). As of July 2023, 37 therapeutic regimens have been studied in over 25,000 patients at hundreds of sites. Both novel and repurposed agents were included, from different classes, with different mechanisms of action, and for both prevention and treatment.

It was imperative to design and deploy trials quickly in order to find and approve treatments that could slow the high mortality and hospitalization rates that persisted throughout the first several years of the pandemic, even though the natural history of COVID-19 was initially unknown. Statisticians and trialists had to grapple with incorporating uncertainty into the designs without undermining the strength of evidence each trial could produce, expecting to face the need for adaptations as new information became available. They leveraged the ACTIV public–private partnership to form open, productive collaborations to solve complex design problems in a short time, advancing the emerging field of platform trials.

Statisticians with prior experience in designing and implementing master protocols and platform trials were part of the group, as were statisticians with extensive infectious disease experience (e.g., from the 2015 Ebola platform trial). Within the first 6 months, three ACTIV master protocols (ACTIV-1, -2, and -3) were developed and launched, and their initial lessons learned have been published [2]. New statistical teams were onboarded until the last protocol was added in April 2021. Throughout this time, the ACTIV Cross-Trial Statistics Group met regularly to discuss problems, share solutions, and educate new team members.

In this manuscript, we describe the varied approaches to trial design and analysis that were incorporated into the 11 ACTIV protocols. We summarize lessons learned in broad design categories in an attempt to advance the science of platform trials and to accelerate responses by the statistical community to future pandemics.

## Coordination of the statistical response

In the early months of the ACTIV initiative, a small group of statisticians from government, industry, and academia was tasked with developing the initial master protocols, meeting frequently in a collaboration characterized by the urgency of the pandemic. Resources, ideas, and expertise were shared openly among this group with no thought as to who would receive credit for the designs or any innovation they represented. On ACTIV-1, for example, analysts from one of the ACTIV industry partners conducted the simulations needed to decide on aggressive futility boundaries, even though the company was not contributing agents to the first study. NIAID statisticians involved with the influential Adaptive COVID-19 Treatment Trials (ACTT-1) shared their data on event rates, ordinal scale distributions, and attrition to facilitate power analyses [3].

As trial networks were identified to lead each master protocol, the group of statisticians expanded. For example, it was decided that ACTIV-2 would leverage the AIDS Clinical Trials Group (ACTG) for implementation, and the ACTG statisticians joined at that time, contributing significantly to developing more than just that one protocol. A senior statistician from the company contributing the first agent to ACTIV-2 joined the working group, sharing in-house data and resources to rapidly advance the protocol.

Weekly meetings of what became known as the ACTIV Cross-Trial Statistics Work Group became critical to advancing knowledge across the 11 master protocols. Each new protocol team brought new statisticians to the group and made design decisions to meet their particular research needs, but robust review of designs (and results) in the group setting helped trial teams to learn from one another, consider the consequences of design choices, and advance the science of platform trials. Feedback from interactions with FDA statisticians were shared, and an FDA statistical reviewer attended meetings, helping with interpretation of review comments and solutions. The ACTIV Cross-Trial Statistics Work Group continued to meet regularly until mid-2023, sharing challenges in operationalizing the trials as well as providing a forum for review and interpretation of results.

*Lessons learned:* When mobilizing for a pandemic, it is critical to provide a forum for statisticians to engage with one another throughout the trial life cycle to optimize the trials for purpose. In particular, the shared perspectives of industry, academia, NIH, other government funding agencies, and regulators, and the willingness to selflessly contribute were pivotal to the rapid response.

## Designing for a pandemic

Trials are traditionally designed in a fairly static context where case rates are constant, the disease trajectory is well characterized, there is consensus in how to measure clinical outcomes, and sample size expectations can be established. Early data in the COVID-19 pandemic were limited to rates of new infections, hospitalization, and mortality, each of which were anticipated to change with new variants and evolution in the standard of care (SoC). Clinical care was expected to change for numerous reasons, including fluctuating demands on the healthcare system, constraints on interacting with infected patients, and the expectation that some interventions being evaluated would emerge as efficacious and be incorporated into SoC. It was also expected that mechanistic understanding would increase over time and result in better risk assessment and inform treatment choices. Incorporating flexibility in the trial designs was key to our ability to react to these challenges of an evolving pandemic.

As the initial ACTIV master protocols were being finalized, results from the early trials of remdesivir were becoming available [3], as were results from the early RECOVERY trials [4,5,6]. ACTIV protocols were therefore generally designed to evaluate new agents as add-on therapy to existing SoC, with allowances for SoC to change during the trial. For example, the ACTIV-1 protocol was originally written with remdesivir as SoC but by the time the study was launched, the RECOVERY study had reported a benefit of dexamethasone [5,7]. At the conclusion of ACTIV-1, nearly as many participants had received dexamethasone as their background medication as had received the originally planned remdesivir alone. ACTIV-6, an outpatient trial, monitors use of background therapy and has shown slow uptake with less than 10% of patients taking monoclonal antibodies, Paxlovid or molnupiravir during 2022 [8]. In addition to changes in SoC over time, there was some variability of SoC choices across sites, but the expectation is that randomized groups remain balanced, supporting valid comparisons for each investigational agent versus placebo.

The desire to be able to discontinue agents early and to add promising new agents to an ongoing trial was reflected in all ACTIV protocols. Some master protocols employed traditional frequentist methods for discontinuation due to futility or early evidence of efficacy. Reflective of the urgency of the pandemic, stopping boundaries for futility were often aggressive. For example, ACTIV-1 required an estimated relative risk ratio for time to recovery of at least 1.05 after approximately 50 percent of the trial’s information was available for an agent to remain in the trial, and ACTIV-3 evaluated futility after 300 participants were enrolled using an intermediate ordinal outcome assessed at study day 5 [9].

Some ACTIV master protocols used a Bayesian statistical framework to address uncertainties in design. ACTIV-2 was designed to evaluate novel therapeutics in outpatients with a seamless Phase II/III trial design. The design incorporated Bayesian decision rules in Phase II, using accumulating data on endpoints such as viral RNA levels or symptoms, to determine if an agent should move to Phase III to assess the clinical endpoint, hospitalization, or death. The ACTIV-4A inpatient protocol evaluated the therapeutic dose of heparin using a fully Bayesian adaptive design. The ACTIV-4A protocol team worked in concert with two other platform trials to prospectively combine all data with a single analysis plan. This multiple platform cooperation allowed for accelerated learning by combining data within the adaptive analysis plan and enabled exploration of heterogeneity of effect by disease severity with this increased sample size. The combined results of three platforms were highly impactful in the global treatment of COVID-19 patients [10,11]. ACTIV-6 studies repurposed medications in the outpatient setting using FDA-recommended endpoints. The statistics team also developed a Bayesian, longitudinal ordinal statistical model to assess the effects of intervention on disease trajectory [12]. The model is intended to rapidly screen out ineffective agents. The ACTIV-4 Host Tissue inpatient platform used Bayesian methods for analysis and decision-making [13]. To reduce time to results, ACTIV-4A added within-person factorial randomization so that participants could be randomized to two treatment modalities (i.e., they could be randomized to receive no drug, one of two study drugs, or both study drugs), decreasing total sample size and speeding up learning about each [14,15]. The wide variety of statistical approaches and designs used in the ACTIV program are described in Table 1.

**Table 1.** Key design characteristics of the ACTIV trials

	ACTIV-1	ACTIV-2*	ACTIV-3	ACTIV-3b	ACTIV-4b: Outpatient	ACTIV4a: In-Hospital	ACTIV-4c: Post-Discharge	ACTIV-4 Host Tissue	ACTIV-5	ACTIV 6
Overview										
Protocol version and date	V2.0 2020-12-02		V5.1 2021-08-17	V3.0, 2022-03-08	V5.7 2021-06-21	V1.2	V5.0 2022-4-20	V5.0 2023-08--07	V7.0 2022-02-10	V10.0 2023-08-02
Patient Population	Inpatient	Outpatient	Inpatient	Inpatient	Outpatient	Inpatient	Outpatient	Inpatient	Inpatient	Outpatient
Phase	Phase III	Phase II-to-III Progressive	Phase III (2 Stage) Progressive	Phase III	Phase III	Phase III	Phase III	Phase III	Arms A/C: Phase II Arm B: Phase II/III	Phase III
Number of study sites (US, ex-US)	85 (95 hospitals), USA and Latin America	173	79 US, 36 ex-US	28 US	52 US	132 US, 35 ex-US	107 US	43 US, 18 ex-US	44 US, 2 ex-US	108 US
Number of participants randomized (all arms)	1971	4044	2752	473	657	3,406	1217	899	820	7742 as of 2023-09-11
Design										
Interventions	Abatacept (single infusion of 10mg/kg with max of 1000 mg), cenicriviroc (300-mg loading dose followed by 150 mg twice per day), or infliximab (single infusion of 5mg/kg). Remdesivir provided as background therapy.	Bamlanivimab (7000 and 700 mg), Amubarvimab/Romlusevimab, Tixagevimab/Cilgavimab (IM and IV), Camostat, SNG001, SAB-185 (low and high doses), BMS-986414+BMS-986413	6 active arms with staggered enrollment: 4 arms nMAbs single infusion: bamlanivimab (7000 mg), sotrovimab (500 mg), BRII-196+BRII-198 (1000 mg), tixagevimab-silgavimab (600 mg); 1 arm DARPin: ensovibep (600 mg, single infusion); 1 arm PI: PF07304814 (IV 250 mg/day for 5 days). Remdesivir provided as background therapy	Aviptadil (3x12 hrs infusions on 3 consecutive days with increasing dose: 600/1200/1800 pmol/kg); remdesivir (200 mg loading dose, followed by 100 mg daily for up to 10 day full course).	Aspirin (81 mg daily), Apixaban (2.5 mg twice daily), Apixaban (5.0 mg twice daily)	Therapeutic heparin, prophylactic heparin, P2Y12 inhibitors, Crizanlizumab, SGLT2 inhibitors	Apixiban 2.5 mg	TXA-127; TRV-027; Fostamatinib	Risankizumab; Lenzilumab, Danicopan. Remdesivir provided as background therapy	Ivermectin at two doses, fluticasone, fluvoxamine at two doses, metformin, and montelukast
Sharing of control group	Shared controls	Shared placebo for all except Bam Phase III (single arm uncontrolled) and SAB-185 Phase III (open-label active comparator)	Shared contemporan-eous controls, except for bamlanivimab & PF07304814 (matched placebo only).	No shared controls	Shared control	Shared control	Not applicable	Shared control	Shared control	Shared controls
Placebo	Agent-specific matched placebo	Agent-specific matched placebo	Agent-specific matched placebo	Agent-specific matched placebo	Placebo (twice daily) (Aspirin arm also included 1 placebo)	Standard of care comparator, open label	Agent-specific matched placebo	Agent-specific matched placebo	Agent-specific matched placebo	Agent-specific matched placebo
Blinding	Double-blind; unblinded for agent	Double-blind with the exception of the non-placebo controlled arms	Double-blind	Double-blind	Double-blind	Open-label; blinded event adjudication	Double-blind	Double-blind; unblinded for agent	Double-blind; unblinded for agent	Double blind; unblinded for agent
Random-ization	2-step randomization; 1:1:1 for agent assignment followed by 3:1 for active vs placebo	2-step randomization using permuted blocks, stratified by days from symptom onset and risk of progression to severe covid	2-step randomization using permuted blocks, stratified by site. Equal allocation to eligible agents, n:1 allocation to active vs matched placebo, where n is the number of active agents.	Randomization using permuted blocks, stratified by design stratum, site, and disease severity	Permuted blocks	Minimization with random component, stratified by inpatient level of care as a surrogate for disease severity	Randomization stratified for antiplatelet use and disease severity	Permuted blocks stratified by study site and trial eligibility	Randomization stratified by site and baseline disease severity	2-step randomization; equal assignment to available interventions followed by n:1 randomization for active vs placebo where *n* is the number of active agents
Interim monitoring	Interim analyses for futility and efficacy at 25, 50%, and 75% of total information. Lan–DeMets spending function for efficacy. Aggressive, nonbinding futility boundaries using the Hwang–Shih–DeCani beta spending function with Gamma (−2).	Monitoring for efficacy and futility	Efficacy monitoring using O’Brien–Fleming boundary with Lan–DeMets alpha-spending function, safety with Haybittle–Peto boundary. Early futility assessment at 300 participants enrolled using a 7-category ordinal outcome assessed at Day 5.	Efficacy and safety using an asymmetrical Haybittle–Peto boundaries. Futility using conditional power.	Monitoring for efficacy, futility, and safety	Interim analyses every 200 participants per domain. Stopping rules applied per domain and subgroup (moderate/severe). Efficacy: P(pOR>1)>0.99; Futility P(pOR<1.2)>0.95; Inferiority: P(pOR<1)>0.99	Trial planned to have interim analyses at 20, 40, 60, and 80% of information content using O’Brien–Fleming rule.	Combined inferiority/futility at 33 and 66% of information content; *ad hoc* conditional power analysis for Fostamatinib	Monitoring for efficacy and futility	Interim analyses planned for futility and efficacy at 25, 50%, and 75% of enrollment into each study drug arm; stopping thresholds based on Bayesian posterior probabilities. Futility based on posterior probability of success
Other adaptive elements	None	Phase II to phase III transition	Tixagevimab-cilgavimab only: planned sample size re-estimation, primary analysis changed to hierarchical testing in full cohort and seronegative subset using Holm’s method.	Planned sample size re-estimation prior to completion of enrollment	None	None	Adaptive changes were planned after Stage 1	None	Unplanned blinded adaptation changed sample size, primary endpoint and population for arm B	Selection of primary endpoint occurs immediately prior to each analysis
Outcomes and analysis										
Primary outcome	Time to recovery by Day 29, where recovery is defined as not hospitalized (regardless of limitations) or hospitalized but not requiring supplement oxygen or ongoing in-patient care (levels 6-8 on 8-point clinical status scale)	Phase II: Safety (grade 3 or higher TEAEs), symptom duration, SARS-COV-2 RNA < LLoQ. Phase III: Safety (grade 3 or higher TEAEs) and all-cause hospitalization or death through 28 days	Sustained clinical recovery through Day 90; early futility assessment using a 7-category ordinal outcome assessed at Day 5.	Six-category ordinal outcome assessed at Day 90. The 6 categories were as follows: (1) at home and off oxygen (recovered) for at least 77 consecutive days by Day 90, (2) at home and off oxygen for 49–76 days, (3) at home and off oxygen for 1–48 days, (4) not hospitalized but either on supplemental oxygen or not at home, (5) hospitalized or in hospice care, or (6) dead.	Composite of death, symptomatic deep vein thrombosis, pulmonary embolism, arterial thrombosis, myocardial infarction, ischemic stroke, hospitalization for cardiovascular or pulmonary events through 45 days after randomization	Organ-support-free days through Day 21	At 30 days, a binary composite endpoint of venous and arterial thrombotic complications – including new, symptomatic proximal, or distal DVT of the upper or lower extremities, PE, and new thrombosis of other veins (including cerebral sinus and splanchnic veins), ischemic stroke, myocardial infarction, other arterial thromboembolism (e.g., mesenteric or acute limb ischemia), and all-cause mortality	Oxygen-free days at Day 28	Arms A/C: Day 8 Ordinal Score Arm B: Mechanical Ventilation-Free Survival through Day 29	Time to sustained recovery (third of three consecutive symptom-free days) OR a composite of 28-day all cause hospitalization or death
Statistical approach	Classical	Classical with Bayesian graduation rules to move agent to Phase III	Classical	Classical	Classical	Bayesian with simulated characterization of Type I error rate	Classical	Bayesian with Type I error control	Classical	Bayesian with approximate Type I error control
Analysis population	ITT for primary analysis; mITT for secondary analyses, defined as all participants receiving at least one dose of the assigned treatment	mITT excluding participants who did not start treatment	mITT excluding participants who did not receive any amount of study agent.	mITT excluding participants who did not receive any amount of study agent.	mITT excluding participants who did not receive any amount of study agent	mITT excluding participants that withdrew before 21 days	ITT, including all randomized participants; secondary analysis used the mITT population excluding those who did not receive study treatment	mITT excluding participants that did not receive study drug or were found to be ineligible after randomization	Arms A/C: mITT excluding participants that did not receive study drug Arm B: ITT in prespecified low-risk subgroup	mITT excluding participants that did not receive study drug or were found to be ineligible after randomization
Statistical model or methodStatistical model or method	Fine-Gray proportional hazards model; estimand is the cumulative incidence of recovery while accounting for the competing risk or death.	Safety assessed using log-binomial; symptom duration by Gehan–Wilcoxon test for TTE; RNA assessed with modified Poisson regression with robust variance; hosp/death assessed as cumulative proportion via Kaplan–Meier, difference in proportions with two-sided Wald CI or exact CI via Chan and Zhang method	Fine-Gray proportional hazards model for time to sustained recovery; proportional odds logistic regression for Day 5 ordinal outcome.	Proportional odds logistic regression model stratified by disease severity.	Risk differences with Newcombe confidence intervals for counts, and log-rank statistics for time to event	Proportional odds logistic regression	A log-binomial regression model with treatment group as an indicator variable and adjustment for WHO ordinal scale and antiplatelet use at baseline	Proportional odds logistic regression	Arms A/C: proportional odds logistic; Arm B: logistic	Proportional hazards regression or logistic regression depending on outcome
Adjustments	Stratification by geographic region and baseline disease severity	RNA models adjusted for baseline RNA, otherwise none	Stratification by site.	Disease severity at baseline.	None	Age, sex, disease severity, and time epoch	Adjusted	Adjusted	Adjusted	Age, sex, duration of symptoms prior to study drug receipt, calendar time, vaccination status, geographical location, call center indicator, and baseline symptom severity
Type I error control	Type I error probabilities controlled by agent for the primary and two secondary endpoints, accounting for interim analyses, using the gatekeeping approach.	Type I error rate controlled by agent on the primary outcome. Hospitalization and death in Phase III adjusted for interim analyses	Type I error controlled by agent; for tixagevimab-cilgavimab, hierarchical test using Holm’s method.	None	None	Simulated characterization of Type I error.	One primary comparison conducted at the Type I error rate of 0.05. A fallback method was used to control Type I error for the ordered secondary endpoints.	Strong familywise control across primary and three key secondary outcomes using the fixed-sequence method	Arm B: Type I error probabilities controlled by agent for the primary and secondary endpoints, accounting for interim analysis, using the gatekeeping approach.	Type I error controlled by use of prespecified, skeptical prior on the treatment effect parameter for the primary outcome

ACTIV-2 reflects two identical master protocols.

*Lessons learned*: A range of statistical approaches that provide flexibility in the presence of uncertainty were available in the early days of the pandemic. As demonstrated by the innovative use of emerging information for decision-making in the ACTIV protocols, the statistical design can and should be tailored to the specific research goals and environment. Interim monitoring of study data is a must, and methods that reduce time-to-decision should be considered so as to rapidly evaluate a large number of potential treatments. Most importantly, having skilled statisticians on hand to select the optimal approach can help ensure that any master protocol is launched with a fit-for-purpose design.

## Endpoint selection

Picking the primary endpoint is one of the most consequential design decisions and may shape the impact of the trial on the medical community. For treatment trials in sicker populations, mortality is a straightforward outcome, and most would agree that reducing mortality is strong evidence in favor of the treatment. Similarly, admission to hospital is straightforward to measure, and with adjudication of the cause of hospitalization, it is not controversial. However, it should be noted that the threshold for hospitalization may change when the number of available hospital beds is limited. In such situations, the criteria for hospitalization may become more stringent, prioritizing patients with the most severe or life-threatening conditions, highlighting the need for concurrent controls.

Early in the pandemic, mortality and hospitalization were considered appropriate trial outcomes in outpatients, assessed using a time-to-event analysis. As ACTIV-3 was designed, time to sustained recovery was considered an important clinical endpoint that would provide acceptable power with a lower sample size than a mortality endpoint, consistent with the remdesivir trial that demonstrated evidence of improved time to recovery but not evidence of improved mortality [3]. Notably, two agents in ACTIV-1 ultimately failed to demonstrate a benefit with time to recovery but did demonstrate a mortality benefit emphasizing the importance of flexibility in the face of emerging knowledge [9].

Increasing the granularity of an outcome increases statistical power. The inpatient ACTIV trials benefitted from the World Health Organization’s clinical status scale as an outcome that quantifies supplemental oxygen support requirements for patients with COVID-19 in addition to hospitalization and mortality [16]. The publication of a common ordinal outcome scale based on vital status and oxygen support, and offering sample size tables for rapid sizing of a trial, accelerated early trial design. Analyzed using ordinal regression methods, the treatment effect can be quantified using a model-based estimand such as the common odds ratio. Perceived drawbacks are as follows:

1. Interpretation of the treatment difference requires additional analyses beyond estimating the common odds ratio to assess the absolute size of the treatment effect across a multi-category outcome. Also, the intensity of public interest in the results of ACTIV clinical trials, particularly for repurposed medications, was unprecedented; anticipating how participants and members of the public will interpret the treatment effect is worthy of consideration.
2. Uncertainty about what time point to choose, and
3. Concern about the assumption of a common odds ratio, although this is mitigated by the fact that the score test for proportional odds regression is equivalent to the nonparametric Wilcoxon–Mann–Whitney statistic, regardless of whether the proportional odds assumption is correct [17,18,19].

Given some of the drawbacks, and in particular concerns about interpretability, the WHO clinical status scale was generally not adopted as the primary outcome for most trials but was used successfully as an early futility outcome (ACTIV-3) and included as a secondary outcome in almost every trial, allowing pooling of patient-level data for cross-trial analyses and direct comparisons among trials. The ACTIV-4a and ACTIV-4 Host Tissue platforms used ordinal endpoints of organ-support-free days and oxygen-free days, respectively, combining mortality and time to recovery into one outcome defined by ordered categories [20]. The common odds ratio in an ordinal regression was used for the estimated clinical effect. As disease severity started to decline, both mortality and hospitalization outcomes declined in frequency. The ACTIV outpatient trials used symptom diaries to assess daily symptoms. The commonly constructed endpoint from these assessments was time to recovery.

It was observed that some patients recover fully, but then had recurrence of symptoms [21]. The ACTIV trials have taken different approaches to measuring time to *sustained* recovery. ACTIV-1 used time to the first of five consecutive days of freedom of symptoms, ACTIV-3 defined “sustained recovery” as being discharged to home and having stayed at home for 14 consecutive days (i.e., the shortest possible time to sustained recovery is 14 days), ACTIV-6 used time to the third of three consecutive symptom-free days, and ACTIV-2 used time to first of two consecutive days of improved symptoms. In addition to different definitions, time to recovery is difficult to interpret in the presence of death and is complicated by hospitalization. To overcome this limitation, ACTIV-6 investigators developed a longitudinal ordinal statistical model and an associated estimand: mean time unwell and days of benefit, both through Day 14. These were intended to provide maximum sensitivity to treatment effect and encode information about bounce back and death. Given the novelty of this endpoint and at the recommendation of the FDA, these were not selected as the primary outcome for the study but instead used as sensitive screening endpoints.

Another observation emerged during the pandemic, which was that some patients were not recovering at all. A constellation of long-term symptoms was being reported, now referred to as post-acute sequelae of COVID (PASC). Several of the trials incorporated longer-term follow-up and secondary endpoints related to prevention and amelioration of PASC. For example, ACTIV-4A added quality of life and 1-year outcomes, and ACTIV-6 extended follow-up to 180 days and added PASC-specific outcomes assessments. Although increasing measurement burden and requiring additional operational support, extending follow-up duration need not require amending the original study design.

The primary endpoint for a trial is a crucial element of trial design and should be selected *a priori*. However, in the fast-paced environment of a pandemic, some flexibility is critical. Like ACTIV-6, ACTIV-5 was originally designed to rapidly screen out ineffective agents; the goal was for the earliest possible assessment of the therapeutic agents and it minimized the sample size per investigational therapy, and leveraged the Day 8 WHO clinical atatus scale as the primary endpoint. In an unplanned adaptation arising from new external efficacy data about one of the agents on the platform, the primary endpoint for that agent was changed mid-study to the binary outcome of mechanical ventilation or death in order to provide evidence of benefit that would be persuasive to regulators. Such change of endpoints has to be initiated and implemented by study personnel blinded to the observed treatment difference, and any statistical calculations would have to be done by blinded statisticians.

There remains interest for other outcomes in inpatient trials, such as days alive and out of hospital or oxygen-support-free days, yet the consistency of use of the WHO scale reflects a broad success of the research community by allowing cross-trial comparisons and measuring the disease with some granularity in its more severe incarnations. Measured longitudinally, the scale also allows for extraction of many of the other outcomes of interests. As understanding of COVID-19 increased, the time frame for observing outcomes also increased, emphasizing the relevance of establishing longitudinal assessments as well as ensuring the inclusion of secondary outcomes that can be broadly compared among trials.

At the time of designing ACTIV-6, it was unclear whether there would be enough mortality or hospitalization events to conduct a feasible trial with these outcomes. In an attempt to avoid unplanned changes to the primary endpoint, the trial team defined two possible primary outcomes – events and recovery – and formally deferred the choice between the two to immediately prior to the specified, scheduled analyses. The choice was made blinded to comparative data from within the trial, but in the presence of external information. The intent was to place the choice of outcome in the context of the pandemic at the time of analysis rather than the time of design. Since those making the decision are blinded to treatment assignment, this does not violate good clinical trial practice. While such fast-changing situations are unusual, we posit that selecting from a predetermined set of endpoints at the time of analysis is reasonable when the disease landscape is changing more rapidly than the evidence generation system. However, the consequences of such an approach on power and sample size should not be ignored. Simulation can be used to evaluate trial operating characteristics under various assumptions. ACTIV-6 demonstrated control of Type 1 error assuming time to recovery would be selected as the efficacy endpoint, and hospitalization and mortality event rates for futility assessment.

*Lessons Learned:* Statistical input to the choice of endpoint is critical, with factors such as ability to ascertain, power, analysis approach, and interpretation of statistical results in terms of clinical meaningfulness at the forefront. Regulatory buy-in is critical, and flexibility must be accommodated. Establishing an efficient process to rapidly select, monitor, and update acceptable endpoints prior to the next pandemic would greatly accelerate trial design decisions and limit unplanned adaptations.

## Multiplicity in master protocols

The issue of multiplicity in platform trials studying several therapeutic agents has been debated on many prominent stages that include regulatory, industry, and academic voices [22,23]. With shared controls, if a Type I error is made on one agent, the conditional probability of a Type 1 error on another agent is increased, despite each agent having controlled marginal Type 1 error [24]. However, the same is true for a gatekeeping procedure in the context of two endpoints. If endpoint 2 is tested only if endpoint 1 is statistically significant, the conditional Type 1 error is substantially higher than 0.05. Nonetheless, gatekeeping procedures are widely accepted because they control familywise error in an unconditional sense. While the conditional rate is increased, we believe the unconditional perspective is more compelling, consistent with Woodcock and LaVange (2016) [25], who note that regulatory decisions about an agent compared to a suitable control are made independently of other agents that may be studied under the same master protocol, and therefore there is no need for a multiplicity adjustment. ACTIV statisticians decided early on that the master protocols would not incorporate multiplicity adjustments for exploring simultaneous arms with shared controls.

*Lessons Learned:* The early involvement of statisticians that were highly facile running platform trials was critical to integrating prior knowledge into the trial design, speeding up development decisions by drawing on specialized clinical trials experience.

## Unexpected design changes

Each of the ACTIV trials platforms was created to address slightly different questions and thus adapted differently in the face of changes. Drivers of change included disease severity, changing case rates, and changing SoC in addition to changes in the underlying immunity of the study population due to vaccination or prior infection. The ACTIV Cross-Trial Statistics Work Group was pivotal to rapid evaluation of likely consequences of design changes, including understanding the regulatory perspective separate from the formal review processes. Several of the platform trials had selected a primary endpoint that became untenable due to changing event rates. For example, in ACTIV 4c, the observed primary clinical event rate and accrual rate were both lower than planned; consequently, a change in the primary endpoint was considered to include a quality of life component (which had substantial missing data). Ultimately, it was decided to merge quality of life outcomes and primary clinical endpoint as a key secondary endpoint. This was because there was no assurance that the change would indeed increase power, and substantial missing data in the quality of life assessments could make the primary results depend on the imputation method used for the missing data.

ACTIV-2 started as a placebo-controlled trial, but as new agents were granted Emergency Use Authorization (EUA), the use of a placebo control became questionable and the platform pivoted to a non-inferiority design comparing the investigational agent to an active control among high-risk people. Design issues related to this included selecting a non-inferiority margin and making assumptions about the likely event rates. The ACTIV-2 team powered their comparisons based on a risk difference instead of a relative risk as the small relative risk would have required an enormous sample size. However, when estimating the sample size required to detect a given absolute risk difference, the trial might be potentially underpowered if the observed control group event rate was lower than assumed. Further complicating matters, as the Omicron variant became widespread, it was discovered that the approved active control agent was not as active against Omicron as it was against prior variants and the FDA favored a return to placebo as the comparator group. The switch was in progress when ACTIV-2 was halted for futility due to low hospitalization and death rates during the Omicron era.

ACTIV-5 was initially a Phase II study in inpatients for all investigated products. However, while ACTIV-5 was enrolling, an external trial demonstrated a treatment benefit of one of the ACTIV-5 agents with a particularly strong result in a *post hoc* subgroup with less severe disease [26]. Given the desire to identify and approve efficacious treatments quickly during the pandemic, the study team modified the ACTIV-5 design for this agent to provide a timely opportunity to confirm the results of the external trial and to potentially enable labeling. In order to expand the trial for the specific agent into a Phase II/III trial, several elements of the study design were changed, including use of a different primary endpoint (occurrence of mechanical ventilation or death through Day 29), larger sample size, addition of conditional power futility analysis, and the target population was restricted to the subgroup with less severe disease. At the recommendation of the FDA, the binary outcome of any occurrence of mechanical ventilation or mortality within 29 days was selected over a time to event analysis as it was felt to be more clinically relevant in this short time frame. The decision for this unplanned adaptation was made by a team who was blinded to any ACTIV-5 interim data by active versus placebo arm.

*Lessons Learned:* While trial analysis plans have to be defined prior to trial execution, changes in external context can result in unplanned adaptations and updates to the statistical analysis plan. A pathway for doing so, blinded to treatment assignment and results, should be available until the conclusion of the trial.

## Shared controls

Because ACTIV master protocols were intended to provide high quality of evidence, including for EUA by the US Food and Drug Administration (FDA), and both novel agents and already-marketed drugs (approved for another indication) would be studied, the gold standard of randomized, double-blind trial design was followed for most trials. This required a matching placebo for every agent tested. This design principle was in contrast with the RECOVERY trials, conducted in the United Kingdom (UK) prior to and in parallel with the ACTIV studies, where treatment assignments were randomized but were open-label [27]. Logistical challenges associated with procuring matched placebos during a pandemic are discussed elsewhere in this thematic issue.

A second design principle adopted from the start was that treatment assignment would be random for both the agent (provided more than one agent was actively being investigated at the time of randomization, and the participant was eligible for more than one agent) and the active agent versus matching placebo. A common implementation method was to employ a two-step randomization, with agent assignment at the firsst step, and active versus placebo assignment at the second step. To allow flexibility with mode of administration, the first step was often unblinded and the second step blinded. That is, participants knew what agent they were randomized to, but not whether it was active or placebo.

With this scheme, it was possible to implement one of the most popular aspects of a master protocol – sharing control patient data across comparative analyses of multiple agents. Because a participant could only be randomized to receive an agent for which they were eligible, data for participants that were assigned to a control group could serve as control for any active agent in that eligibility subset. Controls were only shared across those active arms that were included in step 1 of the randomization (contemporaneous controls). Because of the changing nature of the pandemic, it was decided early on that control data would not be shared across time.

The advantage of sharing a pooled control group across several active agents are the lower overall sample size and thus speedier completion of trials. An illustration is ACTIV-1, where three immune modulators were studied simultaneously (until one was stopped early for futility), and eligibility criteria for the three were very similar. The 1:1:1:1 allocation ratio resulted in a saving of hundreds of participants. Efficiency gains, however, depend on the number of simultaneous treatments being studied at all sites across the trial enrollment period; efficiency gains are less if there are periods when the treatments are not simultaneous and if there are large differences in eligibility criteria. In ACTIV-2, the shared placebo design only achieved an overall sample size reduction of 6%, largely due to complexities in implementing two study phases with different assessment schedules [28].

Pooling control groups should be done with caution. For example, ACTIV-6 observed that treatment response differed between an inhaled and an oral placebo [29]. ACTIV-1, on the other hand, included agents given both orally and by infusion with little impact of that difference on treatment response. There are also logistical challenges, such as the need to maintain blinding within the platform as one of the investigated agents completes and the results are disseminated. Other challenges of multi-arm trials include the possibility of confusing participants during the consent process, as well as the fact that participants may opt out of the study if they are not assigned to an agent they prefer. The need to build firewalls between study teams for individual agents when analyzing an agent in an ongoing protocol that shares controls, the potential introduction of bias when the allocation ratio to active versus control changes with the addition or subtraction of a study arm, and the care required to not publish details about rare events occurring in the shared control arm before all agents finish are just a few examples of the logistical challenges encountered. In general, however, the shared placebo groups in ACTIV protocols provided efficiency gains.

*Lesson learned*: Sharing concurrent control data is recommended when an appropriate design to avoid bias is feasible. In general, we expect the efficiency gains to outweigh the logistical challenges, and careful planning can address the anticipated challenges in advance. That said, sharing controls should not be an automatic design choice; trade-offs between achievable efficiency and complexity for the specific trial context should be considered. Furthermore, sharing controls in a platform trial may impact efficiency gains if there is a differential placebo response; platform trials are encouraged to consider comparability of placebo response before proceeding with a shared control strategy.

## Blinding

The purpose of blinding in a trial is to minimize bias in the estimated treatment effect. All of the ACTIV trials described in Table 1 were blinded to active versus control with the exception of ACTIV-4A, which had blinded assessors. This is in contrast to the RECOVERY trial, which was able to launch rapidly using an open-label approach [27]. There are logistical challenges of setting up and maintaining the blind if using shared controls, as previously noted.

For the ACTIV trials sharing controls and studying multiple agents, participants and providers were made aware which investigational agent they were assigned to but not whether they were assigned to the active or control arm. ACTIV-6 experienced some dropout after participants discovered which agent they were randomized to, suggesting social desirability bias during the consent process. However, in ACTIV-2, ACTIV-3 and ACTIV-4 Host Tissue trials, the dropout rates were low and similar across agents. Key differences included that ACTIV-2, ACTIV-3, and ACTIV-4 Host Tissue studied novel therapies, whereas ACTIV-6 studied repurposed medications, and in ACTIV-6 participants could choose which agents they wished to be considered for while in there was no choice in ACTIV-3 or ACTIV 4 Host Tissue.

*Lessons learned:* Overall, the blinding approaches used for platform trials must consider logistics surrounding shared control participants. The choice to blind may be influenced by pace of implementation, but the benefit of blinding is largely unaffected by pandemic context or uncertainty. Letting participants choose from among available study drug arm arms, or informing them to which study drug arm they were randomized (keeping active versus placebo blinded), can alter willingness to consent and willingness to proceed with trial procedures.

## Other considerations for the statistical analysis plan

As is expected for any trial, the ACTIV investigators finalized statistical analysis plans prior to unblinding. Elements of the plan that require considerable forethought in a platform trial include defining the control group, selection of covariates, and the process for maintaining the blind when extracting control group data. Within a platform, it is preferred to maintain a homogeneous statistical approach, including the handling of missing data and covariate adjustment, which should be prespecified. Similar data collection instruments, data management structures, and consistency in planned statistical analyses across study arms results in substantial efficiencies for support staff, which is particularly important in a pandemic. Nevertheless, we found that within-platform customizations and adaptations were required. Changes included the addition of new covariates based on emerging information about the disease, newly available data such as vaccination, and newly approved SoC therapies. Other contextual changes included declining event rates, requiring updates in sample size considerations. Such changes were documented in protocol appendices, and analysis plans were updated prior to unblinding to ensure any new context was considered in the final analyses. As analysis plans for an individual study arm start to deviate from others on the platform, efficiencies in conducting the analyses diminish and the platform approaches an organizational infrastructure running multiple trials simultaneously.

*Lessons learned:* Selection of a control group, choice of covariates, and the approach to handling changes in context should be prespecified, and changes should be made and documented prior to unblinding. Efficiency gains from using similar data elements and a similar analysis plan for all agents within a platform start to diminish as differences among the individual study arms expand.

## Data and safety monitoring

With expected rapid enrollment in studies evaluating novel agents with uncertain safety profiles in a new disease, frequent safety monitoring was central to the ACTIV trials. Data and Safety Monitoring Boards (DSMBs) were appointed for each trial or set of trials. Given the complexity of the trials, the incorporation of aggressive futility boundaries, and the rapidly changing external context, experienced DSMBs were considered essential. It was recognized there would be considerable burden on the DSMB to review data as frequently as every 2 weeks. Consistency in data collection and monitoring across arms within a platform and consistent layouts of reports were critical to reducing the burden on the DSMBs.

The pacing of DSMB review for a pandemic platform trial has considerable implications. For example, enrollment in both ACTIV-5 and ACTIV-6 far exceeded expectations during the Omicron wave, with thousands of participants enrolled over several weeks during the winter of 2022 in ACTIV-6 alone. Some planned interim analyses could not be implemented because enrollment would have been completed by the time the DSMB could meet, and thus any DSMB recommendation could not be meaningfully actionable.

Beyond activities of a DSMB, monitoring quality of the data is a critical task. Data quality involves a combination of active monitoring and resolution of discrepancies. It is typical not to release trial results until the data are clean and locked. In platform trials during a pandemic, this may not be possible. ACTIV-2, for example, released Day 28 results prior to participant follow-up being completed. With an overwhelming participant enrollment rate in ACTIV-6 and intense pressure to provide information about the treatment effects of drugs being used off label, the study team focused on optimizing the quality of the critical data measured in the first 28 days (eligibility, treatment assignment, and early outcomes). Subsequent evaluation including data out to as long as 180 days means that public use datasets will not mirror precisely the datasets on which the initial analyses were done. While risking criticism of the trial results, this approach reflects the real and practical implications of running trials in a pandemic.

*Lessons learned:* During a pandemic, data and safety monitoring plans should have enough flexibility to accommodate waves in recruitment without sacrificing data quality or participant safety. Data and safety monitoring of platform trials during a pandemic requires considerable analysis and reporting by the study team, a large time commitment of the DSMB members, and an experienced DSMB.

## Data collection, management, and sharing

For rapid trial startup in a pandemic, we used existing, trusted, and tested data infrastructures with the assumption that those infrastructures would be available and sustainable through to final reconciliation of study data. Some existing data management infrastructures were challenged given the need for rapid implementations during the pandemic. There was a need to build in flexibility of the screening system, the randomization system, case report forms, and other ancillary data systems. This was highly resource-intensive as every update or adaptation was an opportunity for error. We also found that during the pandemic, whole new domains of data became critical. For early trials, vaccination was not of concern. Later trials needed to collect vaccination information, and what this meant changed considerably over time. Similarly, collection of medical history and clinical status data items evolved over time. The emergence of long COVID resulted in extension of follow-up windows for newly enrolled patients, and thus different follow-up windows being programmed into the data systems. To add further complexity, many of the ACTIV trials made data collection materials available in multiple languages, potentially delaying deployment of case report forms. Not only do all of the protocol changes need to be reflected in the data system, but updates then need to be translated and the translations approved for deployment, slowing down the opening of new study arms study-wide. With multiple sites open on different versions of a protocol with different agents using multiple languages, complex data management systems are required to deliver the right information to the right stakeholder at the right time. Changes in the data platform occasionally resulted in user error, particularly during the screening and randomization process.

As the pandemic evolved, so did common data elements specific to COVID-19 [30,31,32]. While many of the ACTIV platforms shared case report forms for efficiency, the application of common data elements earlier in the pandemic could have facilitated not only the prospective harmonization of trials within a domain of agents but also DSMB review and secondary and meta-analyses of results. Even in the absence of common data elements, the ACTIV data management systems were designed with the intent of supporting regulatory submissions and for sharing data through the available NIH repositories, and detailed data management plans were implemented in each platform. Evaluating the success of data sharing activities is beyond the scope of this manuscript, barriers to sharing data for ongoing studies in a pandemic have been previously reported [33].

Some of the ACTIV trials were influenced by constraints on biospecimen collection (e.g., blood and nasal swab), slowing down the acquisition of critical pharmacokinetic and virology data. For example, during the pandemic simple procedures for timely shipments of samples to a central biorepository were compromised due to shortages of medical supplies, dry ice, vendors being unable to provide pick-up service, and limited flights for transporting the samples. Collection of biological samples from study participants after discharge from hospital was also challenging. Previously hospitalized participants may still experience significant health issues making transportation difficult. Some may live a significant distance away from the trial site. Discharged patients were also potentially still contagious and hospitals restricted them from returning for in-person study visits. While clinical data can be collected via virtual contact, sampling of biological material requires physical transfer. ACTIV investigators prioritized collection of biological samples and therefore employed a number of workarounds, like setting up specific visit rooms at hospitals, research staff visiting the participant at home, or charging a commercial vendor to do so. While resource-intensive, the investment resulted in excellent adherence to the specimen collection plans.

*Lessons learned:* Appropriate planning for changes, including personnel resources for change management, is critical. Data systems should be configured for flexibility. Data entry logic checks and branching logic can be used to facilitate appropriate targeting of data collection. As well as limiting errors in the enrollment process, workflows should also address variations in site-specific data, such as lab reference ranges and validation limits, to reduce errors in real time. In future pandemics, we recommend careful attention to biospecimen processing so that operational delays to measurements and thus analyses can be addressed early.

## Statistical resources

A common concern among statisticians remains a lack of infrastructure and bandwidth. The ACTIV program benefitted from engaging existing statistical teams working with established networks. Yet, as the master protocols grew with addition of new agents, several issues substantially strained statistical resources:

- Multiple platforms being designed and implemented with limited bench depth in the expertise for designing and deploying platform trials. The Cross-Trial Statistics Work Group was critical to solving this barrier, rapidly educating new members as they joined.
- Rapid pace of reporting to DSMBs.
- The need for each study arm to report to CT.gov independently, including while the platform continues to enroll.
- More than one study arm completing enrollment at the same time resulting in competition for data cleaning and analysis time.
- The accelerated pace of manuscript submissions, with journals requiring additional analyses and changes to data presentation.
- High pressure for rapid completion of secondary and exploratory analyses as the early study arms close.

The time and effort to manage the platform trials was considerable and added to the ongoing demands of redesigning or otherwise amending the previously existing clinical research projects impacted by COVID-19. The strategy of engaging existing statistical teams was highly effective and could be enhanced by establishing access to supportive resources in readiness for a future pandemic response.

*Lessons learned*: A strong recommendation for pandemic preparedness is to pre-identify and maintain statistical resources to guarantee competent leadership and the provision of sufficient statistical support to implement and conduct the trials. In addition to establishing a forum for rapid communication and cross-education, such a resource could include the development of federated data management and analysis models among those critical experienced and trusted partners, with simplified data sharing agreements for use in a pandemic.

## Conclusions

**Figure 1.**
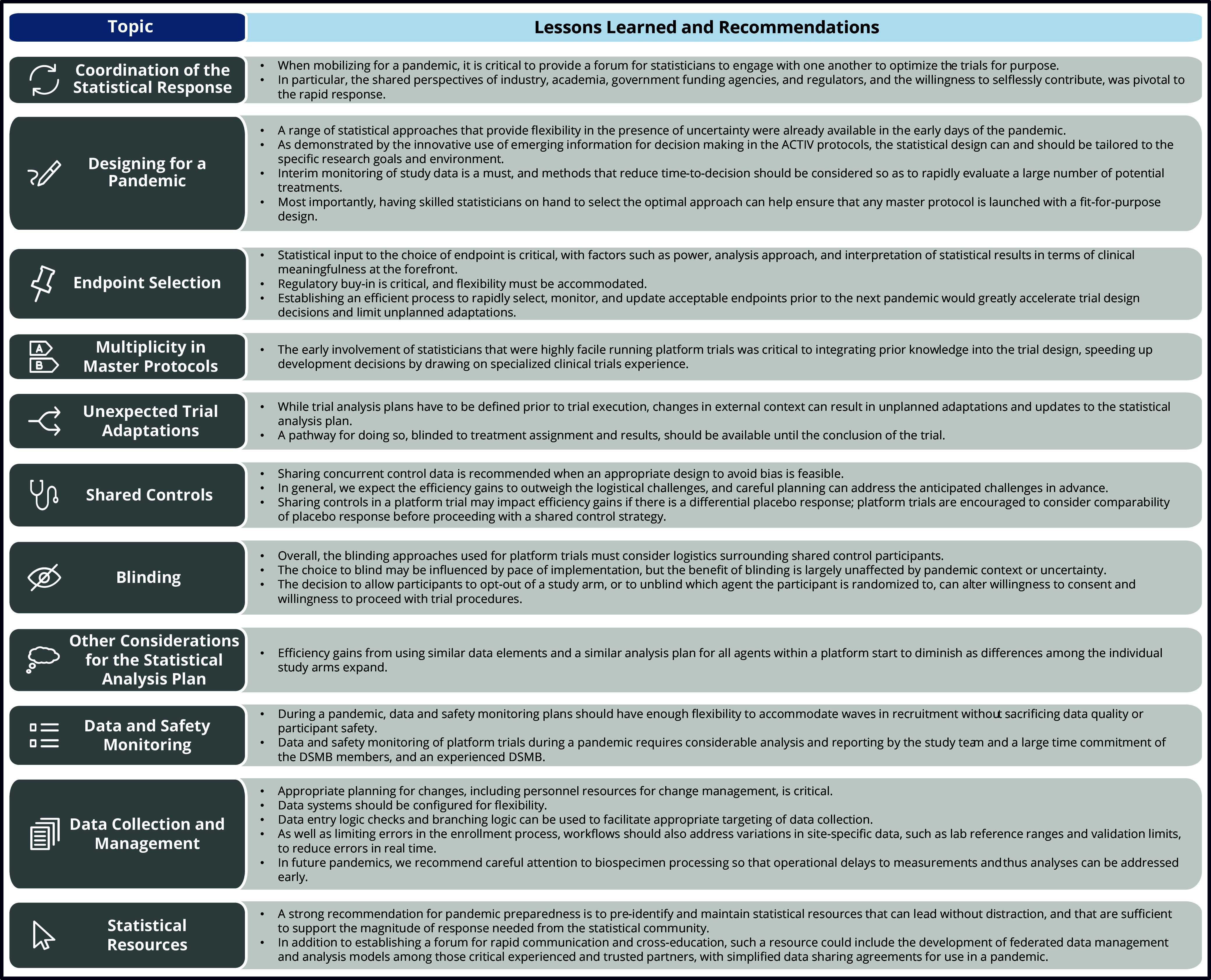
Statistical lessons learned from running platform trials during a pandemic.

In response to the COVID-19 pandemic, the ACTIV initiative launched 11 master protocols in very quick order, at an unprecedented scale. The charge for statisticians was to rapidly design efficient trials with feasible data collection, pivoting as trial adaptations were needed to respond to the evolving pandemic and to support rapid dissemination of study results to improve patient care. Statistical lessons learned are summarized in Figure 1 where we note some practical challenges with applying traditional thinking to platform trials. The call for leveraging existing, well-maintained clinical trial networks to conduct studies in a pandemic should be mirrored with a call to pre-identify several data and statistical centers with sufficient infrastructure, resources, and expertise to support the array of expected trials. We support the overall lessons learned from the ACTIV program that suggest prioritizing fewer, larger master protocols in the next pandemic, specifically three protocols (one for inpatient trials of both novel and repurposed agents, one for outpatient trials with novel agents, and one for outpatient trials with repurposed agents). The Cross-Trial Statistics Work Group mirrored the larger public–private partnership with intense, fruitful, and unselfish collaboration providing peer support and debate to inform design decisions across protocols, and any pandemic playbook should include such a statistical forum.

## References

[1] Sherman RE , Anderson SA , Dal Pan GJ , et al. Real-world evidence - what is it and what can it tell us? New Engl J Med. 2016;375(23):2293–2297. doi: 10.1056/NEJMsb1609216. 27959688

[2] LaVange L , Adam SJ , Currier JS , ACTIV Therapeutics-Clinical Working Group. Accelerating COVID-19 therapeutic interventions and vaccines (ACTIV): designing master protocols for evaluation of candidate COVID-19 therapeutics. Ann Intern Med. 2021;174(9):1293–1300. doi: 10.7326/M21-1269. 34181444 PMC8244665

[3] Beigel JH , Tomashek KM , Dodd LE , ACTT-1 Study Group Members. Remdesivir for the Treatment of Covid-19 - Final Report. N Engl J Med. 2020;383(19):1813–1826. doi: 10.1056/NEJMoa2007764. 32445440 PMC7262788

[4] Horby PW , Mafham M , Bell JL , Linsell L , Staplin N , Emberson J , Palfreeman A , Raw J , Elmahi E , Prudon B , Green C , Carley S , Chadwick D , Davies M , Wise MP , Baillie J K , Chappell LC , Faust SN , Jaki T , Jefferey K , Lim WS , Montgomery A , Rowan K , Juszczak E , Haynes R , Landray MJ. Lopinavir-ritonavir in patients admitted to hospital with COVID-19 (RECOVERY): a randomised, controlled, open-label, platform trial. Lancet. 2020;396(10259):1345–1352. doi: 10.1016/S0140-6736(20)32013-4. 33031764 PMC7535623

[5] Peter H , Wei Shen L , Jonathan E , Group RC. Effect of Dexamethasone in Hospitalized Patients with COVID-19 – Preliminary Report. medRxiv. doi: 10.1101/2020.06.22.20137273.

[6] RECOVERY (2020). Statement from the Chief Investigators of the Randomised Evaluation of COVid-19 thERapY (RECOVERY) Trial on hydroxychloroquine. hcq-recovery-statement-050620-final-002.pdf (recoverytrial.net). Accessed June 5, 2020.

[7] The RECOVERY Collaborative Group. Dexamethasone in hospitalized patients with Covid-19, 2020. N Engl J Med. 2021;384(8):693–704. doi: 10.1056/NEJMoa2021436. 32678530 PMC7383595

[8] Naggie S , Boulware DR , Lindsell CJ , Accelerating Covid-19 Therapeutic Interventions and Vaccines (ACTIV)-6 Study Group and Investigators, et al. Effect of higher-dose ivermectin for 6 days vs placebo on time to sustained recovery in outpatients with COVID-19. JAMA. 2023;329(11):888–897. doi: 10.1001/jama.2023.1650. 36807465 PMC9941969

[9] O’Halloran JA , Ko ER , Anstrom KJ , ACTIV-1 IM Study Group Members, et al. Abatacept, cenicriviroc, or infliximab for treatment of adults hospitalized with Covid-19 pneumonia: a randomized clinical trial. JAMA. 2023;330(4):328–339. doi: 10.1001/jama.2023.11043. 37428480 PMC10334296

[10] Goligher EC , Bradbury CA , McVerry BJ , REMAP-CAP Investigators; ACTIV-4a Investigators; ATTACC Investigators, et al. Therapeutic anticoagulation with heparin in critically ill patients with Covid-19. N Engl J Med. 2021;385(9):777–789. doi: 10.1056/NEJMoa2103417. 34351722 PMC8362592

[11] Lawler PR , Goligher ACTIV-EC , Berger JS , ATTACC Investigators; ACTIV-4a Investigators, et al. Therapeutic anticoagulation with heparin in noncritically ill patients with Covid-19. N Engl J Med. 2021;385(9):790–802. doi: 10.1056/NEJMoa2105911. 34351721 PMC8362594

[12] Naggie S , Boulware DR , Lindsell CJ , et al. Effect of ivermectin vs placebo on time to sustained recovery in outpatients with mild to moderate COVID-19: a randomized clinical trial. JAMA. 2022;328(16):1595–1603. doi: 10.1001/jama.2022.18590. 36269852 PMC9587497

[13] Self WH , Shotwell MS , Gibbs KW , ACTIV-4 Host Tissue Investigators, et al. Renin-angiotensin system modulation with synthetic angiotensin (1-7) and angiotensin ii type 1 receptor–biased ligand in adults with COVID-19. JAMA. 2023;329(14):1170–1182. doi: 10.1001/jama.2023.3546. 37039791 PMC10091180

[14] Jaki T , Vasileiou D. Factorial versus multi-arm multi-stage designs for clinical trials with multiple treatments. Stat Med. 2017;36(4):563–580. 27804166 10.1002/sim.7159 PMC5244690

[15] Solomon SD Lowenstein CJ Bhatt AS , ACTIV4a Investigators, et al. Effect of the P-selectin inhibitor crizanlizumab on survival free of organ support in patients hospitalized for COVID-19: a randomized controlled trial. Circulation. 2023;148:381–390. doi: 10.1161/CIRCULATIONAHA.123.065190. 37356038 PMC10373640

[16] World Health Organization. WHO R&D blueprint: novel coronavirus: COVID-19 therapeutic trial synopsis. https://www.who.int/publications/i/item/covid-19-therapeutic-trial-synopsis. Accessed August 24, 2023.

[17] McCullagh P. Regression models for ordinal data. J R Stat Soc Series B. 1980;42(2):109–142.

[18] Agresti A , Kateri M. Ordinal probability effect measures for group comparisons in multinomial cumulative link models. Biometrics. 2017;73(1):214–219. doi: 10.1111/biom.12565. 27438478

[19] Agresti A , Tarantola C. Simple ways to interpret effects in modeling ordinal categorical data. Stat Neerl. 2018;72(3):210–223. doi: 10.1111/stan.12130.

[20] Moskowitz A , Shotwell MS , Gibbs KW , Fourth Accelerating COVID-19 Therapeutic Interventions and Vaccines (ACTIV-4) Host Tissue Investigators, et al. Oxygen-free days as an outcome measure in clinical trials of therapies for COVID-19 and other causes of new-onset hypoxemia. Chest. 2022;162(4):804–814. 35504307 10.1016/j.chest.2022.04.145 PMC9055785

[21] Chew KW , Moser C , Yeh E , ACTIV-2/A5401 Study Team, et al. Validity and characterization of time to symptom resolution outcome measures in the ACTIV-2/A5401 outpatient COVID-19 treatment trial. J Infect Dis. 2023;228(Suppl 2):S83–S91. doi: 10.1093/infdis/jiad300. 37650237 PMC10469584

[22] EU-PEARL. EU Patient-Centric Clinical Trial Platforms. EU-PEARL’s First Stakeholder Workshop. Multiplicity Framewok. EU-PEARL Presentació OK 2. Accessed October 22, 2020

[23] EU-PEARL. EU Patient-Centric Clinical Trial Platforms. EU-PEARL’s Stakeholder Workshop Site. 2020 Oct 8, 22, 26, and 29. Stakeholder workshop: join us in shapping the future of clinical trials (eu-pearl.eu). Accessed October 8, 2020.

[24] Proschan MA , Follmann DA. Multiple comparisons with control in a single experiment versus separate experiments: why do we feel differently? Am Stat. 1995;49(2):144–149.

[25] Woodcock J , LaVange LM. Master protocols to study multiple therapies, multiple diseases, or both. N Engl J Med. 2017;377(1):62–70. doi: 10.1056/NEJMra1510062. 28679092

[26] Temesgen Z , Burger CD , Baker J , et al. Lenzilumab in hospitalised patients with COVID-19 pneumonia (LIVE-AIR): a phase 3, randomised, placebo-controlled trial. Lancet Respir Med. 2022;10(3):237–246. 34863332 10.1016/S2213-2600(21)00494-X PMC8635458

[27] Randomised Evaluation of COVID-19 Therapy (RECOVERY). RECOVERY Protocol. 2022 May 23. https://www.recoverytrial.net/files/recovery-protocol-v25-0-2022-05-23.pdf. Accessed May 23, 2020

[28] Moser CB , Chew KW , Ritz J , et al. Pooling different placebos as a control group in a randomized platform trial: benefits and challenges from experience in the ACTIV-2 COVID-19 trial. J Infect Dis. 2023;228(Suppl 2):S92–S100. doi: 10.1093/infdis/jiad209. 37650234 PMC10686688

[29] Boulware DR , Lindsell CJ , Stewart TG , ACTIV-6 Study Group and Investigators, et al. Inhaled fluticasone furoate for outpatient treatment of COVID-19. N Engl J Med. 2023;389(12):1085–1095. doi: 10.1056/NEJMoa2209421. 37733308 PMC10597427

[30] Weissman A , Cheng A , Mainor A , et al. Development and implementation of the national heart, lung, and blood institute COVID-19 common data elements. J Clin Transl Sci. 2022;6(1):e142. 36590348 10.1017/cts.2022.466 PMC9794959

[31] Edlow BL , Boly M , Chou SH , et al. Common data elements for COVID-19 neuroimaging: a GCS-neuroCOVID proposal. Neurocrit Care. 2021;34(2):365–370. doi: 10.1007/s12028-021-01192-6. 33575956 PMC7878171

[32] Admon AJ , Wander PL , Iwashyna TJ , et al. Consensus elements for observational research on COVID-19-related long-term outcomes. Medicine. 2022;101(46):e31248. 36401423 10.1097/MD.0000000000031248 PMC9678399

[33] Palm ME , Lindsell CJ , Selker HP. Sharing data among clinical trials of therapeutics in COVID-19: barriers and facilitators to collaborating in a crisis. J Clin Transl Sci. 2022;6(1):e52. 35599687 10.1017/cts.2021.866 PMC9114727

