# The statistical design and analysis of pandemic platform trials: Implications for the future – CORRIGENDUM

**DOI:** 10.1017/cts.2024.648

**Published:** 2024-10-28

**Authors:** Christopher J. Lindsell, Matthew Shotwell, Kevin J. Anstrom, Scott Berry, Erica Brittain, Frank E. Harrell, Nancy Geller, Birgit Grund, Michael D. Hughes, Prasanna Jagannathan, Eric Leifer, Carlee B. Moser, Karen L. Price, Michael Proschan, Thomas Stewart, Sonia Thomas, Giota Touloumi, Lisa LaVange

The original article listed Carlee B. Moser as Carly B. Moser. This has been amended in the original publication.
